# Genomic content of a novel yeast species *Hanseniaspora gamundiae* sp. nov. from fungal stromata (*Cyttaria*) associated with a unique fermented beverage in Andean Patagonia, Argentina

**DOI:** 10.1371/journal.pone.0210792

**Published:** 2019-01-30

**Authors:** Neža Čadež, Nicolas Bellora, Ricardo Ulloa, Chris Todd Hittinger, Diego Libkind

**Affiliations:** 1 Biotechnical Faculty, University of Ljubljana, Jamnikarjeva 101, Ljubljana, Slovenia; 2 Laboratorio de Microbiología Aplicada y Biotecnología, Instituto de Investigaciones en Biodiversidad y Medio-ambiente, Consejo Nacional de Investigaciones, Científicas y Técnicas (CONICET)-Universidad Nacional del Comahue, Bariloche, Argentina; 3 Laboratorio de Bioprocesos, Instituto de Investigación y Desarrollo en Ingeniería de Procesos, Biotecnología y Energías Alternativas, Consejo Nacional de Investigaciones, Científicas y Técnicas (CONICET)-Universidad Nacional del Comahue, Neuquén, Argentina; 4 Laboratory of Genetics, Genome Center of Wisconsin, DOE Great Lakes Bioenergy Research Center, Wisconsin Energy Institute, J. F. Crow Institute for the Study of Evolution, University of Wisconsin-Madison, Madison, Wisconsin, United States of America; Leibniz Institut - Deutsche Sammlung von Mikroorganismen und Zellkulturen GmbH, GERMANY

## Abstract

A novel yeast species was isolated from the sugar-rich stromata of *Cyttaria hariotii* collected from two different *Nothofagus* tree species in the Andean forests of Patagonia, Argentina. Phylogenetic analyses of the concatenated sequence of the rRNA gene sequences and the protein-coding genes for actin and translational elongation factor-1α indicated that the novel species belongs to the genus *Hanseniaspora*. *De novo* genome assembly of the strain CRUB 1928^T^ yielded a 10.2-Mbp genome assembly predicted to encode 4452 protein-coding genes. The genome sequence data were compared to the genomes of other *Hanseniaspora* species using three different methods, an alignment-free distance measure, *K*_r_, and two model-based estimations of DNA-DNA homology values, of which all provided indicative values to delineate species of *Hanseniaspora*. Given its potential role in a rare indigenous alcoholic beverage in which yeasts ferment sugars extracted from the stromata of *Cytarria* sp., we searched for the genes that may suggest adaptation of novel *Hanseniaspora* species to fermenting communities. The *SSU1*-like gene encoding a sulfite efflux pump, which, among *Hanseniaspora*, is present only in close relatives to the new species, was detected and analyzed, suggesting that this gene might be one factor that characterizes this novel species. We also discuss several candidate genes that likely underlie the physiological traits used for traditional taxonomic identification. Based on these results, a novel yeast species with the name *Hanseniaspora gamundiae* sp. nov. is proposed with CRUB 1928^T^ (ex-types: ZIM 2545^T^ = NRRL Y-63793^T^ = PYCC 7262^T^; MycoBank number MB 824091) as the type strain. Furthermore, we propose the transfer of the *Kloeckera* species, *K*. *hatyaiensis*, *K*. *lindneri* and *K*. *taiwanica* to the genus *Hanseniaspora* as *Hanseniaspora hatyaiensis* comb. nov. (MB 828569), *Hanseniaspora lindneri* comb. nov. (MB 828566) and *Hanseniaspora taiwanica* comb. nov. (MB 828567).

## Introduction

Since the introduction of DNA sequence analysis for species delineation, the number of newly described species of the yeast genera *Hanseniaspora* and *Kloeckera* (the allied anamorphic genus) has increased from seven to nineteen [1–6]. A similar number of newly described species can be observed for other yeast genera, mostly as an extensive database of barcode sequences of D1/D2 and internal transcribed spacer (ITS) regions provides data of all described species [7]. However, additional protein-coding genes have also been employed in yeast taxonomy to construct statistically well-supported phylogenetic trees that reflect the evolutionary relationships among species and genera [8–10]. With emergence of whole genome sequencing, reconstructions of more robust yeast phylogenies are now being recovered [11–14], from which a frame for new species definition is being built [15–21]. A shift from phenotype- to sequence-based taxonomy enables introduction of the new *International Code of Nomenclature for algae*, *fungi*, *and plants* (Shenzhen Code; [22]) by which the teleomorphic genus *Hanseniaspora* has a priority over its anamorphic counterpart, *Kloeckera*, by the rules of nomenclature and the number of species assigned to each genus [23]. Accordingly, the three asexual species *Kloeckera hatyaiensis*, *Kloeckera lindneri* and *Kloeckera taiwanica* should be transferred to the genus *Hanseniaspora* (see Taxonomy).

Ecologically, yeasts belonging to the genus *Hanseniaspora* are the most common of the apiculate yeasts. They are found on various fruits, flowers, and bark as their primary habitat. Insects may also serve as their dispersal vectors, and the soil may serve as their reservoir [24,25]. The main sources of simple sugars in the environment include the decaying fruits on which *Hanseniaspora* species predominate and fruiting bodies, such as those of *Cyttaria hariotii*, a fungal parasite of *Nothofagus*, a tree endemic to Southern hemisphere forests. During maturation, *C*. *hariotii* stromata become rich in fermentable sugars, a substrate typical for yeast communities in which *Hanseniaspora* species often becomes prevalent [16,26–28]. However, the availability of simple sugars from ripe fruits is short-term, which is suggesting that apiculate yeasts mostly reside in soil or are associated with other plant material [29]. For *Hanseniaspora* species, these habitats are also consistent with their assimilation profiles because they can utilize only those few carbon sources that are available in tree bark, such as cellobiose, arbutin, and salicin [30]. Additionally, *Hanseniaspora* yeasts have unusually high vitamin requirements [31], which further suggests their close association with plant material.

During the exploration of the biodiversity of fermenting yeast communities in the stromata of the tree fungus *C*. *hariotii* in Andean Patagonia [27,32–33], we found two apiculate yeast strains that were distinct from known species. DNA sequence comparisons of rRNA- and protein-coding genes suggested that they represent a novel yeast species, which is described here as *Hanseniaspora gamundiae* sp. nov. We also provide a draft genome sequence and analysis of *Hanseniaspora gamundiae* CRUB 1928^T^ to accompany the formal description of the new species. Interestingly, genome content correlates with several physiological traits and suggests possible adaptations of this species to its ecological niche.

## Materials and methods

### Yeast isolation and physiological characterization

The two strains examined in this study were isolated from the stromata of *C*. *hariotii* of *Nothofagus dombeyi* (“Coihue”) and of *Nothofagus antarctica* collected from the Andean forests of Patagonia, Argentina (S1 Table). The Argentinean National Park Administration issued a permission for sampling. Stromata were processed as described by Libkind et al. [27] and Sampaio and Gonçalves [34], respectively. Strain CRUB 1604 was isolated using selective media of Yeast Nitrogen Base (YNB, Difco) supplemented with 1% (w/v) raffinose and 8% (v/v) ethanol and purified on Yeast Malt Agar (YMA). Strain CRUB 1928^T^ was isolated without any carbon source in the media, although pieces of *C*. *stromata*, which naturally contain simple sugars, were added as an inoculum. The strains were phenotypically characterized by standard methods, including assimilation tests conducted in liquid media for 3 weeks at 26°C [35]. Sporulation was investigated on 5% malt agar (Difco; Becton, Dickinson and Company) at 26°C over 3 weeks.

### Phylogenetic placement

The MasterPure Yeast DNA Purification kit (Epicentre) was used to extract DNA from cultures grown on yeast extract-peptone-glucose agar (YPD, Sigma) plates for 2 days. Amplification of DNA sequences encoding the internal transcribed spacer (ITS, 657 bp), the large subunit rRNA (LSU) D1/D2 domain (571 bp), actin (encoded by *ACT1*, 949 bp), and translation elongation factor-1a (EF-1α, encoded by *TEF1*, 695 bp) was performed as described by Cadez *et al*. [36]. These DNA sequences were determined by a commercial sequencing facility (Macrogen Inc., The Netherlands).

The dataset of ITS-D1/D2-actin-EF-1α sequences generated during this study, along with the previously determined sequences of the related species [2], were aligned using the MUSCLE algorithm [37] and concatenated. Phylogenetic relationships were inferred by the Maximum Likelihood (ML) method using the substitution model of Tamura-Nei [38] with gamma-distribution rates (G) calculated in MEGA6 [39]. Bootstrap support [40] was determined from 1,000 pseudoreplicates. Conflicting topologies of single gene ML trees were determined by using Consensus Network algorithm (threshold 0.1) as implemented in program SplitsTree 4.14 [41]. Parsimony network analysis was performed using the aligned ITS-D1/D2 sequences, excluding gaps at 95% connection limit with the program TCS 1.21 [42].

### PCR fingerprinting

To examine the genetic relatedness between the proposed new species and its closest relatives, genomic fingerprinting using three microsatellite primers ((ATG)_5_, (GTG)_5_, and (GACA)_4_) was used in PCR amplification reactions, as described previously [43]. Similarities between the combined fingerprints were calculated using the Pearson’s product moment correlation coefficient (r), based on the overall densitometric profiles of the banding patterns. Cluster analysis of the pairwise values was performed using the UPGMA algorithm as implemented in the BioNumerics 7.5 computer program.

### Preparation of library for sequencing genomic DNA

For genome sequencing, *Hanseniaspora gamundiae* strain CRUB 1928^T^ was cultured in 15 ml YM broth [35] for 72 h at 26°C. DNA was extracted as described by Bellora et al. [44]. About 5 μg of gDNA was sonicated and ligated to Solexa sequencing adaptors (Illumina) using the manufacturer’s kit [45]. The paired-end library was sequenced by using the Illumina HiSeq 2000 and MiSeq in accordance with the manufacturer’s instructions.

### Genome assembly and annotation

*De novo* genome assembly was performed with SPAdes 3.9.0 [46] with options -m 60 -t 20. A total of 6,841,738 paired-end reads from five sequencing sets with mean lengths of 145, 96, 95, 96, and 94 nt and estimated insert sizes of 369, 332, 356, 163, and 333 nt, respectively, yielded 6722 contigs. These contigs were merged into 338 scaffolds longer than 2000 bp with median read coverage of 29.3. Read coverage was used to estimate the ratio between single-copy nuclear, mitochondrial, and rDNA regions. Reads were aligned to the ITS/5.8S region, D1/D2 region, *ACT1*, and *TEF1* of the *Hanseniaspora gamundiae* sp. nov. CRUB 1928^T^ genome with Blat v34 [47] using the default parameters (NCBI accession numbers KU674841, KU674853, KU674865, and KU674877, respectively).

Simple repeats and transposable elements were identified by RepeatMasker v4.0.3 [48] using the RepBase database [49]. Only those repetitive elements with more than 100 occurrences were considered. tRNAs were predicted by tRNAscan-SE v1.23 [50]. GeneMark-ES v2.3e [51] was used for gene prediction (parameters: -min_contig 8000 –max_nnn 1000). Coding sequences (CDSs) were retrieved by recording the Reciprocal BLAST Best Hits (e-value < 10^−5^, identity > 50%) against the *Saccharomyces cerevisiae* (SGD) proteome [52] as a reference and to the proteome of the *Hanseniaspora vineae* T02-19AF (NCBI) genome predicted with GeneMark-ES v2.3e [51] using the same parameters as above. All predicted genes were annotated by using Blast2GO 3.3 [53].

### Phylogenomic analyses

Similarities between the genomes were calculated using an alignment-free matching algorithm with a DNA seed based on 9 bases (110110101000111), as implemented in BioNumerics 7.6. Subsequent clustering using these similarity values enabled phylogenomic placement of *H*. *gamundiae*. The level of genomic sequence divergence between closely related species was estimated using the *K*_r_ value, an alignment-free pairwise distance measure calculated with Genome Tools [54]. The calculations of Average Nucleotide Identity (ANI) values were performed by web-based calculator available at https://www.ezbiocloud.net/tools/ani [55]. We also estimated DNA–DNA homology (DDH) values with the Genome-to-Genome Distance Calculator (GGDC) 2.1 provided by the DSMZ web site (http://ggdc.dsmz.de/distcalc2.php). The DDH values presented here were calculated using Formula 2, which estimates DDH values based on the identities of high-scoring segment pairs (HSPs) [56].

### Assigning genes to enzyme functions

Predicted protein sequences were assigned an enzyme function using TBLASTN and BLASTP searches using query protein sequences from the characterized pathways in the model organism *Saccharomyces cerevisiae* S288c against subject databases built from the *Hanseniaspora* genome assemblies. We used Best reciprocal hits (BRH) relying on BLASTP hits (e-value threshold of 10^−5^) to estimate the presence of each gene.

### Nomenclature

The electronic version of this article in Portable Document Format (PDF) in a work with an ISSN or ISBN will represent a published work according to the International Code of Nomenclature for algae, fungi, and plants, and hence the new names contained in the electronic publication of a PLOS article are effectively published under that Code from the electronic edition alone, so there is no longer any need to provide printed copies.

In addition, new names contained in this work have been submitted to MycoBank from where they will be made available to the Global Names Index. The unique MycoBank number can be resolved and the associated information viewed through any standard web browser by appending the MycoBank number contained in this publication to the prefix http://www.mycobank.org/MB/. The online version of this work is archived and available from the following digital repositories: PubMed Central, LOCKSS.

## Results and discussion

### Species delineation and phylogenetic placement

**Fig 1:**
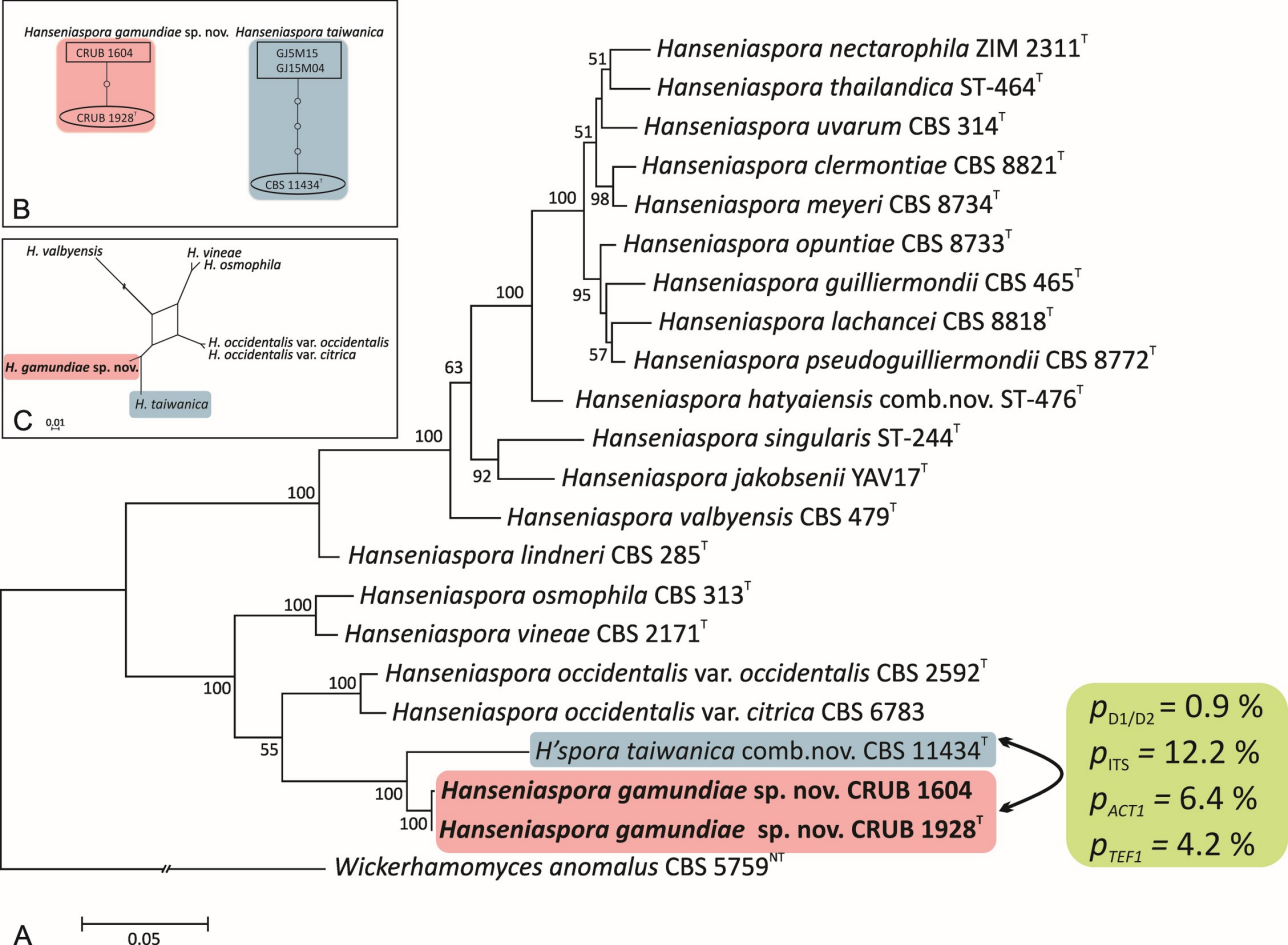
Placement of *Hanseniaspora gamundiae* sp. nov. within the genus *Hanseniaspora* based on the sequences of the ITS and the D1/D2 LSU regions of the gene encoding the rRNA, as well as the genes encoding actin and elongation factor-1α (EF-1α) proteins. (A) A phylogenetic tree inferred using Maximum-Likelihood analysis based on the Nei-Tamura model in MEGA6 [39]. Bootstrap analysis was carried out with 1000 pseudoreplicates [40]; only values above 50% are shown. *Wickerhamomyces anomalus* was used as the outgroup. Bar, nucleotide substitutions per site. *p*, indicating percentage of substitutions between the two marked sequences for each of the four individual genes or *p-*distance. (B) Parsimony networks based on the ITS and D1/D2 LSU of the rRNA gene sequences of strains of *H*. *gamundiae* sp. nov. and its closest relative *H*. *taiwanica*. Each connecting line represents a single nucleotide substitution, and each small circle indicates a missing intermediate haplotype. The rectangle indicates the sequences identified as ancestral by the analysis. Analyses were performed using TCS 1.21 [42]. (C) A consensus network showing relationships between *H*. *gamunidae* and its closest relatives. *H*. *mollemarum* [6] is not included in phylogenetic analyses.

While studying the diversity of native fermenting yeasts from natural Andean forests in Patagonia, Argentina, in particular that of *Saccharomyces* spp., we studied approx. 600 samples of bark, soil and *Cyttaria* stromata along all Andean Patagonia [16,32–33]. The frequency of yeasts in those samples averaged 70%, of which ca. 54% were *Saccharomyces* spp. either *S*. *eubayanus* or *S*. *uvarum* [33]. Other frequent species were *Kregervanrija* spp., *Kregervanrija delftensis*, *Torulaspora* af. *delbrueckii*, *Torulaspora microellipsoides*, *Zygosaccharomyces cidrii*, *Lachancea nothofagi*. Among those were two apiculate yeast strains (S1 Table). We sequenced and analyzed the genes encoding the ITS/5.8S and LSU D1/D2 domains of rRNA and the actin and translation elongation factor-1α (EF-1α) proteins. The two isolates shared identical D1/D2 LSU and actin gene sequences, while they differed by two nucleotides each in the ITS and in the EF-1α datasets (strains CRUB 1604 and CRUB 1928^T^). A BLAST similarity search with the D1/D2 LSU revealed that the apiculate yeasts belonged to the genus *Hanseniaspora* and that these sequences did not have an exact match in the GenBank. Their nearest match was to the type strain of *H*. *taiwanica* (EF653942) with 5 substitutions. This phylogenetic placement was also statistically well supported (bootstrap 100%) by phylogenetic analysis of the concatenated gene sequences encoding ribosomal rRNA, actin, and EF-1α (Fig 1A). Because the classical guidelines for species delineation introduced by Kurtzman and Robnett [57] may be overly conservative for closely related *Hanseniaspora* species [36], we identified interspecific discontinuity by parsimony haplotype network analysis of the concatenated ITS and D1/D2 sequences, as suggested by Lachance *et al*. [58]. At the 95% connection limits, haplotypes of the two Argentinian strains investigated in this study formed a parsimony network separated from *H*. *taiwanica* (69 missing links in 1210 bp dataset), indicating the genetic isolation of both taxa (Fig 1B). This differentiation was further confirmed by pairwise comparisons between strain CRUB 1928^T^ of the new species and the neighboring type strain of *H*. *taiwanica* in their D1/D2, ITS, *ACT1*, and *TEF1* datasets. As shown in Fig 1A, the percentage of sequence divergences in protein-coding genes (*ACT1* and *TEF1*) exceeded 3%, which we consider indicative of two closely related but distinct species of *Hanseniaspora* [1]. However, the pairwise distances of 12.2% in the ITS region differed widely from the pairwise distances of 0.9% in the LSU D1/D2 region between Argentinian strains and *H*. *taiwanica* (Fig 1A). These findings further confirm heterogeneities in the evolutionary rates of genetic regions; compared to the majority of ascomycetous yeasts, ITS regions evolve unusually quickly, while D1/D2 regions are unusually conserved in *Hanseniaspora* species [36,57]. As such conflicting signal can lead to a less accurate phylogeny [11, 59] we reconstructed the relationship between *H*. *gamundiae* and its closest relatives also in form of a phylogenetic network (Fig 1C). It confirmed previously determined relationships (Fig 1A) between these species excluding presence of conflicting phylogenetic signals. Nevertheless, the restricted number of the strains studied may hamper the estimation on intraspecific variation.

The genetic divergence of the strains belonging to the novel species was confirmed by PCR fingerprinting as well. The PCR-fingerprints of the strains CRUB 1604 and CRUB 1928^T^ segregated them from their closest relatives (S1 Fig). Based on the data presented above, we propose a novel yeast species, *Hanseniaspora gamundiae* sp. nov. (MycoBank no. MB824091), to accommodate these strains within the genus *Hanseniaspora*.

### Genome analysis

*De novo* genome assembly of *Hanseniaspora gamundiae* type strain CRUB 1928^T^ yielded a 10.2-Mbp genome assembly with a coverage of 29.3-fold, which was assembled into 338 scaffolds with shortest scaffold at the 50% of genome length of 63 kb, and with 0.46% of unknown bases (Table 1).

**Table 1:** Comparative analysis of genomes, assemblies, and gene statistics for *Hanseniaspora gamundiae* sp. nov. and other *Hanseniaspora* species.

		Size (Mb)	Scaffolds	L50	N50 (kb)	%N	GC content	Genes	Accession No.
***H*. *gamundiae* sp. nov.**	CRUB 1928^T^	10.2	338	45	63	0.46	37.1%	4,452	PTXO00000000
***H*. *vineae***	T02-19AF	11.4	305	40	83	0	37.4%	4,733	JFAV02000000
***H*. *osmophila***	AWRI 3579	11.5	17	4	1,273	0.77	37.1%	4,657	LPNM01000000
***H*. *uvarum***	34–9	8.1	105	13	142	0	32.0%	n.a.	JPPO02000000
***H*. *uvarum***	DSM 2768	9.5	335	11	251	0	32.6%	3,043	APLS01000000
***H*. *uvarum***	AWRI 3580	8.8	18	3	1,289	0.03	31.9%	4,061	LPNN01000000
***H*. *opuntiae***	AWRI 3578	8.8	17	3	1,278	0.06	35.1%	4,167	LPNL01000000
***H*. *guilliermondii***	UTAD222	9.0	208	32	91	0.03	30.9%	4,070	FQNF01000000
***H*. *valbyensis***	NRRL Y-1626	11.4	647	11	332	15.51	26.7%	4,772	LXPE00000000

n.a., not available data; L50, the smallest number of scaffolds whose length sum produces N50; N50, the sequence length of the shortest scaffold at 50% of the total genome length; %N, percentage of unknown bases.

The G+C content of the new species’ genome is 37.1%, which is similar to the genomes of *H*. *vineae* [60] and *H*. *osmophila* [61]. The latter two species have the highest G+C values known for *Hanseniaspora* species, whether measured by exact calculations from the genome sequencing (Table 2) or estimated by thermal denaturation (an approach whose estimates were generally from 1.5 to 3% higher [62]). Of the 4,624 predicted genes, 4,452 were cannonical protein-coding genes (96.3%, Table 2). As introduced by Bellora et al. [44], by using the excess sequencing coverage of contigs for the mitochondrial genome (mtDNA) and *rDNA* loci, we estimated the copy number ratio of mtDNA per nDNA as 883:1 and the *rDNA* locus per nDNA as 77:1 in the genome of *H*. *gamundiae* CRUB 1928^T^ (Table 2).

**Table 2:** Genome statistics.

Attribute	Value	% of total
Genome size (bp)	10,183,153	100%
DNA coding region (bp)		
DNA G+C content (bp)	3,759,798	37.09%
Total genes	4624	100%
Protein-coding genes	4452	96.3% of total genes
*rDNA* locus	77	-
*tRNA* genes	43	-
mtDNA : nDNA ratio	770 to 997:1	-
Repeats (bp)	273,933	2.7% of genome size
Class I: Retrotransposons	1099	9.3% of repeats
LTR transposons	595	5.0% of repeats
Non-LTR transposons (DIRS, LINE)	504	4.2% of repeats
Class II: DNA transposons	1121	9.4% of repeats
Simple repeats	8130	68.5% of repeats
A and GA-rich regions	1216	10.2% of repeats
Other	301	2.5% of repeats
Genes with function prediction	4128	89.3% of total genes
without function prediction	496	10.7% of total genes
Both *H*. *vineae* and *S*. *cerevisiae* orthologs	3451	74.6% of genes with function predicted
Only *Hanseniaspora vineae* orthologs	368	8.0% of genes with function predicted
Only *Saccharomyces cerevisiae* orthologs	309	6.7% of genes with function predicted

LTR, long terminal repeat; DIRS, *Dictyostelium* intermediate repeat sequence; LINE, long interspersed nuclear element.

Next, the phylogenetic markers LSU D1/D2, ITS/5.8S, EF-1α, and actin (KU674858, KU674846, KU674882K, and KU674870, respectively) were aligned to the genome sequences of the same strain. The number of mismatches in genome assembly was low as there was only one to two nucleotide differences in sequences determined by Sanger sequencing. Repeat and low complexity sequences cover 2.7% of the genome and, of those, 27% are represented by transposable elements (TEs). TEs present in more than 100 copies per genome were classified [63] as LTR retrotransposons corresponding to *Gypsy*/*Ty3* and *Copia*/*Ty1*, *LINE* elements corresponding to *Jockey* and *L1* of non-LTR retrotransposons, and *hAT* superfamilies corresponding to DNA cut-and-paste transposons. Most of these elements are widespread in the family Saccharomycetaceae [64].

Annotated genes of *H*. *gamundiae* were mapped against *de novo*-predicted genes of *H*. *vineae* T02/19AF and genes of *S*. *cerevisiae* S288c from Saccharomyces Genome Database using the reciprocal best BLAST hit algorithm. The majority of the 3451 genes (74.6%) were shared by all three species, and only a small proportion was specific to either the closely related *H*. *vineae* or the distantly related *S*. *cerevisiae*: 8% and 6.7%, respectively (Table 2). Recently, Shen et al. [13] reported on the low coverage of *H*. *valbyensis* and *H*. *uvarum* genes (62.2% and 64.5%, respectively) from the BUSCO set of 1438 single-copy, conserved fungal genes, suggesting accelerated evolutionary rates or pervasive gene loss in these genomes. Nevertheless, for *H*. *vineae*, which is closely related to *H*. *gamundiae* but more distantly related to *H*. *valbyensis* and *H*. *uvarum*, this phenomenon was not observed. For *H*. *gamundiae*, we found 242 out of the 248 highly conserved set of core eukaryotic genes (CEG) proposed by Parra *et al*. [65], suggesting that *H*. *gamundiae* is also not a victim of accelerated evolution or pervasive gene loss.

### Genomic diversity

**Fig 2:**
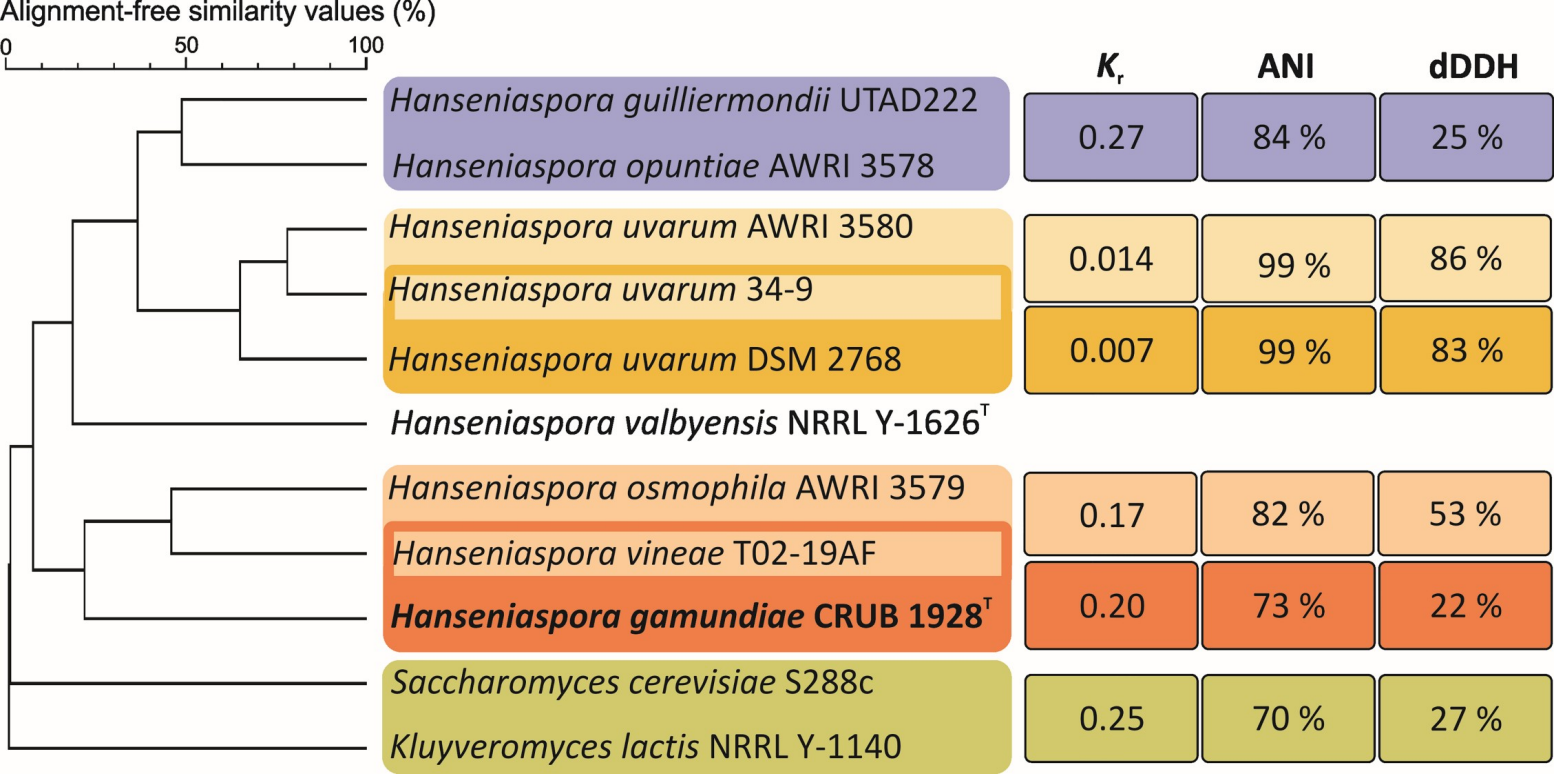
Phylogenomic relationships of the nine *Hanseniaspora* strains based on an alignment-free matching algorithm with a nine bases DNA seed. *Saccharomyces cerevisiae* and *Kluyveromyces lactis* were used as outgroups. Similarities among closely related genomes are presented as the Alignment-Free Distance Measure (*K*_r_), Average Nucleotide Identity (ANI) and by estimating digital DNA-DNA Homology values (dDDH values).

The genome of *H*. *gamundiae* CRUB 1928^T^ was compared to the genomes of six out of nineteen *Hanseniaspora* species (Table 1) and to distantly related species of the family Saccharomycetaceae using an alignment-free matching algorithm. As shown in Fig 2 and S2 Table, the species placement agreed with the tree based on the concatenated ribosomal RNA and protein-coding genes (Fig 1).

The genome similarities among closely related species were estimated using an alignment-free distance measure, *K*_r_ [54]. *H*. *gamundiae* and its closest sequenced relative, *H*. *vineae*, share *K*_r_ values of 0.2, a value previously shown by Bellora et al. to correspond to distinct species [44]. However, the *K*_r_ values are somewhat lower for the comparison of the closely related taxa of *H*. *vineae* and *H*. *osmophila*, which share intermediate DNA-DNA reassociation values (45–60%, [62]). Since boundaries between *Hanseniaspora* species were originally set using DNA-DNA reassociation measurements, we estimated DNA-DNA homology values (DDH) between *Hanseniaspora* genomes by using Average Nucleotide Identity (ANI) calculator [55] and Genome-to-Genome Distance Calculator (GGDC, [56]). The highest scores using ANI calculator were among closely relates species pairs *H*. *guilliermundii-H*. *opuntiae* and *H*. *vineae-H*. *osmophila* which is in agreement with previous conclusions based on DNA-DNA reassociation values of 35% and 33%, respectively [36,62]. However, the dDDH values were higher only among the closely related *H*. *vineae-H*. *osmophila* species pair (53%). On the other hand, *H*. *gamundiae* had lower similarity scores (19.7%-30%) when compared to all other *Hanseniaspora* genomes. Both approaches proved useful for providing indicative values to delineate species of *Hanseniaspora*.

### Habitat and possible adaptation to fermenting communities

Both strains of the novel species *Hanseniaspora gamundiae* were isolated during our survey of fermentative yeasts colonizing obligate biotrophic stromata of *Cyttaria* spp. (Ascomycota, Leotiomycetes, Cyttariales) and the bark of its host trees, *Nothofagus* spp., suggesting the new yeast species is associated with this niche. Its closest known relative is the recently described species of *H*. *taiwanica* [4], whose three strains were isolated from fruiting bodies of different mushrooms in Taiwan. These are the only reports of isolations of *Hanseniaspora* species from mushrooms, making it difficult to infer them as their fundamental niche. Standard physiological profiles for these species also do not reveal a clear picture. The main carbon sources found in stromata are the sugar alcohol mannitol, the disaccharide trehalose, and the polysaccharides glycogen and chitin [66], none of which can be assimilated by either *H*. *gamundiae* or *H*. *taiwanica* suggesting their dependence on other community members [67]. However, the sugar composition of mature *C*. *hariotii* consists of up to 10.2% of the simple sugars of fructose, glucose, and sucrose [68,69], which resembles the composition of grape juice, a substrate in which *Hanseniaspora* species predominate.

The Mapuche tribe, whose traditional territory is coincident with the *Nothofagus* forests in Patagonia, collected and consumed *Cyttaria* stromata in many ways, some of which continue to the present [70]. Similar to the many cereals, fruits, and roots fermented by people elsewhere, natives of this tribe used this fungus to produce an alcoholic fermented beverage (called *chicha)* from these sugar-rich stromata [71]. The *Cyttaria* beverage could have been obtained by squeezing fresh stromata and collecting the resulting juice, which fermented spontaneously, or simply by leaving the entire stromata in cooled boiled water for a few days [72]. Several yeasts inhabit *Cyttaria* stromata in their various maturation stages [16,28], although most are not likely to have contributed much to the distinctive properties of *chicha*. Given that both strains of *H*. *gamundiae* were isolated in the late maturation stages of stromata as part of a fermenting microbiota and given that *Hanseniaspora* spp. play a significant role in sugar fermentation and flavor generation during wine production [73,74], we speculate that *H*. *gamundiae* could have been a major player in the initial stages of traditional *chicha* fermentation. Much as occurs during the spontaneous wine fermentation with communities rich in *S*. *cerevisiae*, we further speculate that *chicha* fermentations may have been traditionally outcompeted at later stages by *Saccharomyces uvarum* and/or *Saccharomyces eubayanus* [16,27,28].

In this context, we searched the genome sequence of *H*. *gamundiae* for genes that may provide advantages during mixed species fermentation. One such gene encodes a sulfite efflux pump (*SSU1*); among *Hanseniaspora* species with available genome sequences, *SSU1* is present only in *H*. *gamundiae* and its closest relatives, *H*. *vineae* and *H*. *osmophila*, (Table 1). *Saccharomyces* spp. produce sulfite in the presence of fermentable sugars and under nitrogen-limiting conditions, and this compound arrests the growth of sulfite-sensitive yeasts in mixed communities [75,76]. However, the functionality of *SSU1* was not verified in this study.

**Fig 3:**
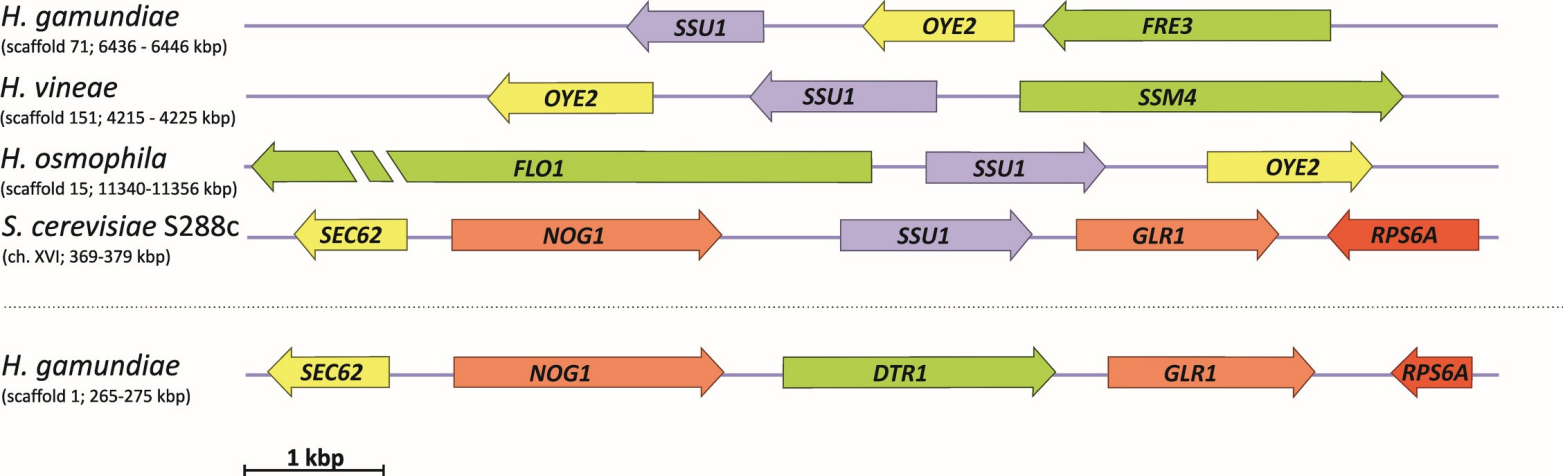
Schematic gene organization around the putative sulfite pump gene *SSU1* (purple) in *H*. *gamundiae* sp. nov. and related species. Green arrows indicate genes without synteny, while the color intensity of arrows from yellow (40–50% nucleotide similarity), to orange (80–90% similarity), to red (more than 90% similarity), indicate the level of conservation between orthologous genes. Gene sizes and distances are approximately to scale. Numbers in parentheses indicate scaffold number and location of genes in the assembled genome.

Detailed investigation of the organization of the *SSU1* locus in *Hanseniaspora* spp. (Fig 3) revealed that the *SSU1* gene is adjacent to a gene encoding a NAPDH dehydrogenase (*OYE2*-like), whose *S*. *cerevisiae* homolog is involved in oxidative stress response. This arrangement is reminiscent of the linkage between *SSU1* and *GLR1*, which encodes a glutathione reductase, in the genome of *S*. *cerevisiae* [77,78]. Interestingly, compared to *S*. *cerevisiae*, *H*. *gamundiae* has a conserved gene order and an unusually high sequence identity of those genes that are in proximity of the *SSU1* gene. However, instead of a gene encoding a sulfite efflux pump, it harbors the *DTR1* gene, which encodes a putative dityrosine transporter that is expressed during sporulation [79]. Similar synteny conservation has also been reported for some representatives of the family Saccharomycetaceae, such as *Eremothecium assybii*, *Kluyveromyces lactis*, and a group of European *Saccharomyces paradoxus* strains that harbor a second copy of *SSU1* of unknown origin next to *DTR1* [80,81]. During the diversification of *Hanseniaspora*, genomic rearrangements may have resulted in different positions for the *SSU1* gene within the genome, but its conservation may have been key for the success of some *Hanseniaspora* spp. in habitats like the sugar-rich stromata of *Cytarria* that are rich in fermenting competitors. Nevertheless, due to the seasonal occurrence of mature *Cyttaria* stromata, they might represent only a transient habitat for *H*. *gamundiae*, and *SSU1* conservation may also be important in surrounding niches, including immature *Cyttaria*, insects, tree bark, or the soil underneath trees.

### Physiological characteristics of *H*. *gamundiae* and their correlation to the genome content

Similar to other *Hanseniaspora* species, *H*. *gamundiae* is characterized by a limited set of growth abilities, which precludes the use of conventional physiological tests for its identification. *H*. *gamundiae* can be distinguished from its sibling species, *H*. *taiwanica*, only by its inability to assimilate glucono δ-lactone, while it can be distinguished from *Hanseniaspora occidentalis* only by its maximum growth temperature.

**Fig 4:**
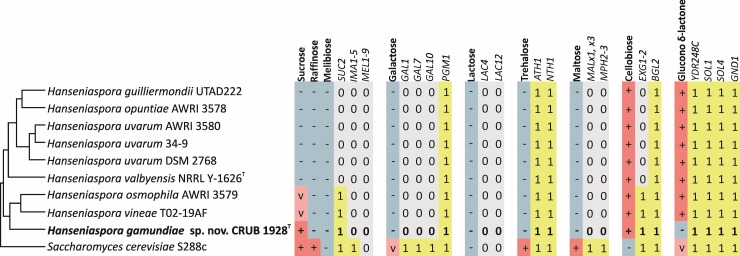
Metabolic traits and genes for the assimilation of the carbon sources sucrose, raffinose, melibiose, galactose, lactose, trehalose, maltose, cellobiose, and glucono δ-lactone by *Hanseniaspora* species and *Saccharomyce cerevisiae* (S288c). Numbered boxes indicate presence (1) or absence (0) of putative homologous genes determined by Reciprocal Best Hit (RBH) prediction algorithm. Physiological characteristics are from Kurtzman *et al*. [84], except for *H*. *gamundiae*.

In addition, we searched the genome for candidate genes underlying the physiological traits used for traditional identification in order to understand the genetic causes of yeasts metabolic diversity [20,82]. We analyzed the genome content of *H*. *gamundiae* and the publically available *Hanseniaspora*’s genomes for the presence of genes required for the utilization of disaccharides, as well as raffinose and glucono δ-lactone (Fig 4). As species of *Hanseniaspora* can assimilate only a limited number of carbon sources, they also mostly lack homologs of the genes required for their utilization. This fact partly explains the low number of ORFs in the genome of *H*. *valbyensis* predicted by Riley *et al*. [82] in a comparative study of yeasts belonging to subphylum Saccharomycotina. The only exception is presence of homologs of genes encoding predicted acid trehalase (*ATH1*) and neutral trehalase (*NTH1*), which are required for utilization of extracellular trehalose [83], a sugar on which none of the analyzed *Hanseniaspora* species can grow. Since these genes are also involved in the metabolism of trehalose manufactured inside the cell, it is possible that these genes play a role in the stress response or non-catabolic processes, instead.

One of the few sugars that *H*. *gamundiae* can assimilate and weakly ferment is sucrose, and this ability is strain-specific in its close relatives *H*. *vineae* and *H*. *osmophila* [84]. Unfortunately, no phenotypic data exists for *H*. *vineae* T02-19AF and *H*. *osmophila* AWRI 3579, the strains whose genome sequences are available [60,61]. Nevertheless, based on our genome sequence analyses (Fig 4), it seems likely that the presence of the *SUC2* homolog encoding invertase is required for sucrose utilization in *Hanseniaspora*.

In contrast to the other *Hanseniaspora* species with available genomes, *H*. *gamundiae* is unable to assimilate glucono δ-lactone. In *Saccharomyces cerevisiae*, three enzymes are believed to be involved in metabolism of glucono δ-lactone [85] namely 6-phosphogluconolactonase encoded by *SOL1* and *SOL3*, 6-phosphogluconate dehydrogenase encoded by *GND1*, and gluconokinase which might be encoded by the ORF *YDR248C*. We found that, even though it cannot consume the carbon source, *H*. *gamundiae* has homologs of all four genes that are thought to be essential for the growth on glucono δ-lactone. This discordance between phenotype and genotype in *H*. *gamundiae* is an additional example that metabolic traits of non-conventional yeasts cannot always be inferred from model organisms [20,86].

**Fig 5:**
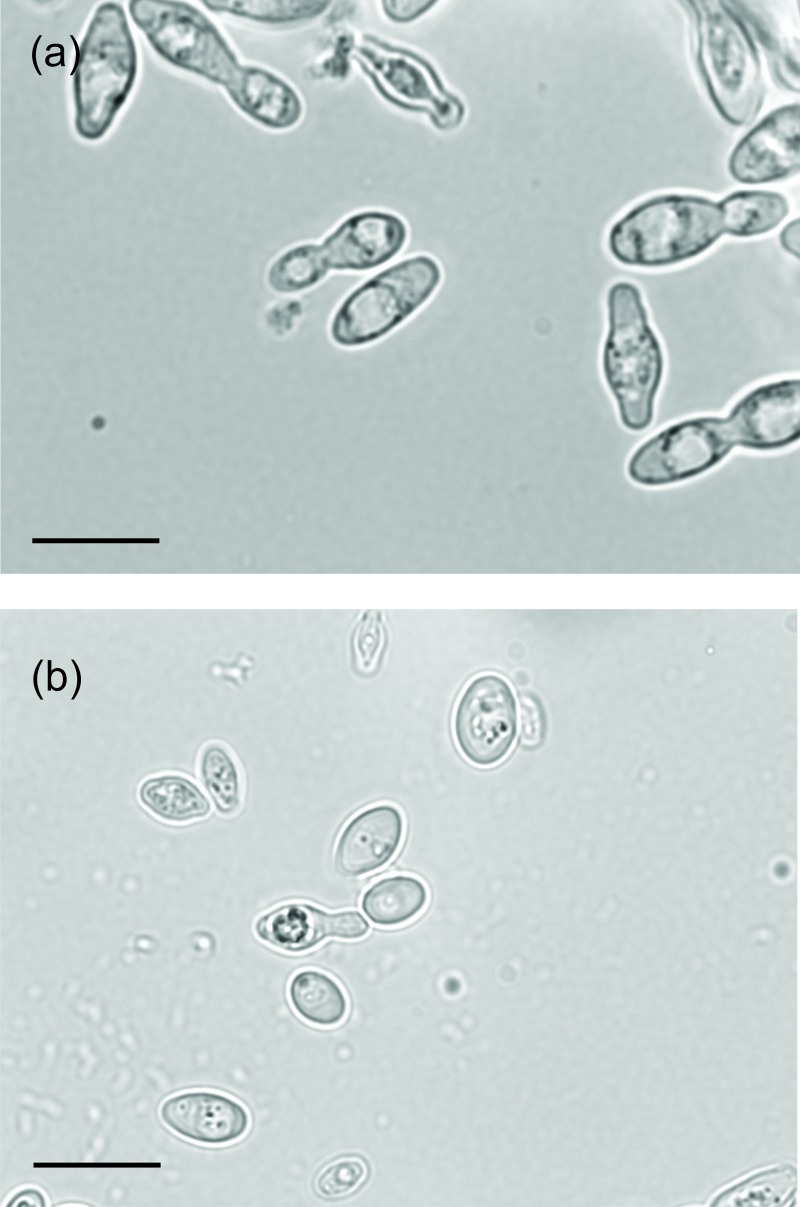
*Hanseniapora gamundiae* sp. nov. CRUB 1928^T^. (a) Budding cells, YM broth, 25°C, 220 rpm, 2 days. (b) Sphaerical and warty ascospore, 5% malt extract agar after 21 days at 25°C. Scale bars, 10 μm.

Morphologically both strains of *H*. *gamundiae* produced one to two sphaerical and warty ascospores (Fig 5), a feature shared with *H*. *vineae* and *H*. *osmophila* and not to its close relative of *H*. *occidentalis*. Strain CRUB 1604 is characterized by slow growth rate on standard yeast growth media (e.g. Yeast Malt Agar).

### Taxonomy

#### Description of *Hanseniaspora gamundiae* Libkind, Čadež, Hittinger 2018, sp. nov. [Mycobank accession: MB824091]

Etymology: Specific epithet *gamundiae* (N.L. gen. fem. N.), “of Gamundi”, to honour Dr. Irma Gamundi (Argentina) in recognition for her valuable contributions on fungal systematics, particularly in the genus *Cyttaria*.

Standard description: The species belong to the genus *Hanseniaspora* in the Saccharomycetales. On YM agar, after 1 month at 25°C, the steak culture is cream colored, butyrous, smooth, glossy, and flat to slightly raised at the center, with an entire to slightly undulate margin. On slide culture with cornmeal agar, a rudimentary pseudomycelium is formed. In yeast extract-malt extract liquid medium after 48 h at 25°C, the cells are apiculate, ovoid, or elongate (4.3–15.7) μm × (2.4–4.7) μm; they occur singly or in pairs. Budding is bipolar. Sediment is present, and a very thin ring is formed after 1 month. Asci containing one to two sphaerical and warty ascospores (1.4–4.3 μm) are observed after 2 weeks or more on 5% malt extract agar and YM agar at 26°C. Ascospores are mostly not released from the ascus (Fig 2). Glucose and sucrose (weakly) are fermented; D-galactose, maltose, lactose, and cellobiose are not fermented. The carbon compounds that are assimilated are glucose, sucrose, cellobiose, salicin, and arbutin; no growth occurs on galactose, L-sorbose, D-glucosamine, *N*-acetyl-D-glucosamine, D-ribose, D-xylose, L-arabinose, D-arabinose, L-rhamnose, maltose, α,α-trehalose, methyl α-D-glucoside, melibiose, lactose, raffinose, melezitose, inulin, starch, glycerol, erythritol, ribitol, xylitol, L-arabinitol, D-glucitol, D-mannitol, galactitol, *myo*-inositol, glucono-δ-lactone, 2-keto-D-gluconate, D-gluconate, D-glucuronate, D-galacturonate, DL-lactate, succinate, citrate, methanol, ethanol, propane-1,2-diol, butane-2,3-diol, and hexadecane. Assimilation of nitrogen compounds is positive for ethylamine hydrochloride, lysine, and cadaverine, while it is negative for potassium nitrate, sodium nitrite, creatine, creatinine, glucosamine, and imidazole. Growth in vitamin-free medium is absent. Growth occurs at 30°C but is absent at 35°C. Growth with 10% NaCl is positive, but growth is absent with 16% NaCl, on 50% (w/w) glucose-yeast extract agar, with 1% acetic acid, and with 0.01% cycloheximide. The diazonium blue B reaction is negative. Unambiguous identification and phylogenetic placement is based on DNA sequences of the following nuclear loci: ITS/5.8S (KU674846), D1/D2 (KU674858), *ACT1* (KU674870) and *TEF1* (KU674882). The species habitats are *Cyttaria hariotii* stromata infecting *Nothofagus* spp. trees in Argentina.

The holotype CRUB 1928^T^ was isolated from *Cyttaria hariotii* stromata infecting *Nothofagus dombeyi* “Coihue” collected at Perez Rosales pass, Patagonia, Argentina in spring 2007 and is preserved in a metabolically inactive state in the Centro Regional Universitario Bariloche Yeast Culture Collection, Argentina. Ex-type cultures are deposited at the Collection of Industrial Microorganisms, Slovenia (ZIM 2545^T^), ARS Culture Collection, National Center for Agricultural Utilization Research, IL, USA (NRRL Y-63793^T^), Portuguese Yeast Culture Collection, Portugal (PYCC 7262^T^) and at the University of Wisconsin-Madison, WI, USA (yHCT65^T^). The Mycobank number is MB824091. The BioProject number for raw genome sequencing reads is PRJNA434570 (BioSample SAMN08564278), and the GenBank accession number for the assembled genome is PTXO00000000.

A shift from phenotype- to sequence-based taxonomy enables introduction of the new International Code of Nomenclature for algae, fungi, and plants (Shenzhen Code; [22]) by which the teleomorphic genus *Hanseniaspora* has a priority over its anamorphic counterpart, *Kloeckera*, by the rules of nomenclature and the number of species assigned to each genus [23]. Accordingly, we propose the transfer of three *Kloeckera* species to the genus *Hanseniaspora*.

#### Description of *Hanseniaspora hatyaiensis* (Jindamorakot, Ninomiya, Limtong, Kawasaki & Nakase) Čadež & Libkind f.a., comb. nov. [MB 828569]

Basionym: *Kloeckera hatyaiensis* Jindamorakot, Ninomiya, Limtong, Kawasaki & Nakase (2009). FEMS Yeast Res 9: 1327–1337. [MB 514510]

Holotype ST-476; Ex-type cultures: BCC 14939, NBRC 104215, CBS 10842.

#### Description of *Hanseniaspora lindneri* (Klöcker) Čadež & Libkind f.a., comb. nov. [MB 828566]

Basionym: *Pseudosaccharomyces lindneri Klöcker* (1912). Zentralbl. Bakteriol. Parasitenkd., Abt. II, 35: 375–388. [MB 180162]

≡ *Kloeckera lindneri* (Klöcker) Janke (1928). Zentralbl. Bakteriol. Parasitenkd., Abt. II, 76: 161. [MB 467907]

Holotype CBS 285; Ex-type culture: NRRL Y-17531.

#### Description of *Hanseniaspora taiwanica* (Lee) Čadež & Libkind f.a., comb. nov. [MB 828567]

Basionym: *Kloeckera taiwanica* C.F. Lee (2012), Int J Syst Evol Microbiol 62:1434–1437. [MB 561891]

Holotype EJ7M09; Ex-type cultures: BCRC 23182, CBS 11434.

## Supporting information

S1 Table List of the strains studied, their origins, and their GenBank accession numbers. (PDF)

S2 Table Similarities among closely related genomes. The values are presented as the Alignment-Free Distance Measure (Kr), Average Nucleotide Identity (ANI) and by estimating digital DNA-DNA Homology values (dDDH values) in comparison to DNA-DNA reassociation [36,62] where available.
(PDF)

S1 Fig UPGMA dendrogram based on PCR fingerprints obtained with the microsatellite primers (GTG)5x and (ATG)5x on *Hanseniaspora gamundiae* sp. nov. and their closest relatives. The distances between the strains were computed using Pearson's correlation coefficient.
(PDF)
