# Supplementary material for: Genomic content of a novel yeast species Hanseniaspora gamundiae sp. nov. from fungal stromata (Cyttaria) associated with a unique fermented beverage in Andean Patagonia, Argentina
Source: PLoS One. 2019 Jan 30;14(1):e0210792. doi: 10.1371/journal.pone.0210792 (PMC6353571; doi:10.1371/journal.pone.0210792)
Supplement: S1 Table — (PDF) [file pone.0210792.s001.pdf]

S1 Table. List of the strains studied, their origins, and their GenBank accession numbers.

Strain accession number*, †	Origin	GenBank accession numbers‡			
		ITS/5.8S	***D1/D2***	***ACT1***	***TEF1***
Hanseniaspora gamundiae sp. nov.					
CRUB 1928^T, isolate SR10-K1 (=ZIM 2545^T = NRRL Y-63793^T = PYCC 7262^T = yHCT65^T)	*Cyttaria hariotii*, *Nothofagus dombeyi*, Perez Rosales pass, Patagonia, Argentina	KU674846	KU674858	KU674870	KU674882
CRUB 1604, isolate RH69	*Cyttaria hariotii*, *Nothofagus antarctica*, Rucahuenu, Patagonia, Argentina	KU674841	KU674853	KU674865	KU674877
Hanseniaspora clermontiae					
CBS 8821^T	Stem rot, *Clermontia* sp., Hawaii	AJ512441	AJ512452	AM039472	AM039516
Hanseniaspora guilliermondii					
CBS 465^T	Infected nail, South Africa	AJ512433	U84230	AM039457	AM039501
Hanseniaspora hatyaiensis comb. nov.					
ST-476^T (BCC 14939^T)	Rotten wood, Songkhla Province, Thailand	KF958035	DQ404528	KF958085	KF958110
Hanseniaspora lachancei					
CBS 8818^T	Fermenting agave juice, Mexico	AJ512439	AJ512457	AM039469	AM039513
Hanseniaspora lindneri					
CBS 285^T	Soil, Java	AJ512430	U84226	AM039454	AM039498
Hanseniaspora meyeri					
CBS 8734^T	Fruit of *Sapindus* sp., Hawaii	AJ512436	AJ512454	AM039466	AM039510
Hanseniaspora nectarophila					
ZIM 2311^T	Flower of *Siphocampylus corymbiferus,* Brazil	KF958047	KF958072	KF958095	KF958120
Hanseniaspora occidentalis var. *occidentalis*					
CBS 2592^T	Soil, St. Croix, West Indies	AJ512429	U84225	AM039463	AM039507
Hanseniaspora occidentalis var. *citrica*					
CBS 6783	Orange juice, Italy	AJ973092	AJ973101	AM039464	AM039508
Hanseniaspora opuntiae					
CBS 8733^T	*Opuntia ficus-indica* rot, Hawaii	AJ512435	AJ512453	AM039465	AM039509
Hanseniaspora osmophila					
CBS 313^T	Ripe Riesling grape, Germany	AJ512431	U84228	AM039455	AM039499
Hanseniaspora pseudoguilliermondii					
CBS 8772^T	Orange juice concentrate, Georgia, USA	AJ512437	AJ512455	AM039467	AM039511
Hanseniaspora singularis					
ST-244^T (BCC 15001^T)	Flower, Narathiwat Province, Thailand	KF980889	FJ391977	KF958094	KF958119

Hanseniaspora taiwanica comb. nov					
CBS 11434^T	*Russula* sp. fruiting body, Hsinchu Country, Taiwan	FJ873604	EF653942	KF958102	KF958127
Hanseniaspora thailandica					
ST-464^T (BCC 14938^T)	Insect frass, Narathiwat Province, Thailand	AB501148	DQ404527	KF958103	KF958128
Hanseniaspora uvarum					
CBS 314^T	Muscatel grape, Crimea, Russia	AJ512432	U84229	AM039456	AM039500
Hanseniaspora valbyensis					
CBS 479^T	Soil, Germany	AJ512434	U73596	AM039458	AM039502
Hanseniaspora vineae					
CBS 2171^T	Soil of vineyard, South Africa	AJ512443	U84224	AM039459	AM039503

* CRUB, Centro Regional Universitario, Argentina, ZIM, Collection of Industrial Microorganisms, Slovenia, NRRL, ARS Culture Collection, National Center for Agricultural Utilization Research, IL, USA, PYCC Portuguese Yeast Culture Collection, Portugal, BCC, BIOTEC Culture Collection, Thailand.

† T, type strain

‡ ITS/5.8S, internal transcribed spacer region/ 5.8S rRNA gene; D1/D2, LSU D1/D2 domain of rRNA gene; *ACT1*, gene encoding actin; EF-1α, translation elongation factor-1α encoded by the *TEF1* gene.

nd, not determined
