# Supplementary material for: Genomic content of a novel yeast species Hanseniaspora gamundiae sp. nov. from fungal stromata (Cyttaria) associated with a unique fermented beverage in Andean Patagonia, Argentina
Source: PLoS One. 2019 Jan 30;14(1):e0210792. doi: 10.1371/journal.pone.0210792 (PMC6353571; doi:10.1371/journal.pone.0210792)
Supplement: S2 Table — The values are presented as the Alignment-Free Distance Measure (Kr), Average Nucleotide Identity (ANI) and by estimating digital DNA-DNA Homology values (dDDH values) in comparison to DNA-DNA reassociation [36,62] where available. (PDF) [file pone.0210792.s002.pdf]

S2 Table: Similarities among closely related genomes. The values are presented as the Alignment-Free Distance Measure (Kr), Average Nucleotide Identity (ANI) and by estimating digital DNA-DNA Homology values (dDDH values) in comparison to DNA-DNA homology values (DDH) [35,62] where available.

	H.gam				*H.vin*				*H.osm*				*H.val*				*H.uva* 34-9			
	K_r	dDDH	ANI	DDH	K_r	dDDH	ANI	DDH	K_r	dDDH	ANI	DDH	K_r	dDDH	ANI	DDH	K_r	dDDH	ANI	DDH
H.gam																				
H.vin	0.20	22%	73%	nd																
H.osm	0.28	25%	73%	nd	0.17	53%	82%	48%												
H.val	0.30	21%	72%	nd	0.30	22%	72%	28%	0.30	23%	72%	24%								
H.uva 34-9	0.28	21%	72%	nd	0.25	21%	71%		0.30	22%	71%		0.30	24%	75%					
H.uva AWRI	0.29	21%	71%	nd		21%	72%	9%	0.30	22%	72%	12%	0.30	24%	75%	20%	0.014	86%	99%	nd
H.uva DSM	0.29	30%	71%	nd	0.30	32%	72%		0.30	33%	71%		0.30	26%	75%		0.007	83%	99%	
H.gui		21%	71%	nd		22%	71%	12%		22%	71%	13%		24%	75%	22%		24%	78%	
H.opu	0.30	21%	71%	nd	0.30	22%	71%	6%	0.30	23%	71%	15%	0.30	25%	75%	17%	0.30	24%	77%	
K.lac	0.26	24%	70%	nd	0.26	25%	71%	nd	0.30	25%	70%	nd	0.30	21%	71%	nd	0.30	22%	70%	
S.cer	0.30	24%	70%	nd	0.30	24%		nd	0.30	26%	70%	nd	0.30	21%	71%	nd	0.30	23%	71%	

	H.uva AWRI 3580				*H.uva* DSM 2768				*H.gui*				*H. opu*				*K.lac*			
	K_r	dDDH	ANI	DDH	K_r	dDDH	ANI	DDH	K_r	dDDH	ANI	DDH	K_r	dDDH	ANI	DDH	K_r	dDDH	ANI	DDH
H.gam																				
H.vin																				
H.osm																				
H.val																				
H.uva 34-9																				
H.uva AWRI																				
H.uva DSM	0.011	77%	99%	nd																
H.gui		24%	78%	nd		26%	77%	nd												
H.opu	0.30	24%	78%	nd	0.30	27%	77%	nd	0.27	25%	84%	35%								
K.lac	0.30	22%	70%	nd	0.30	32%	70%	nd	0.30	24%	70%	nd	0.30	24%	70%	nd				
S.cer	0.30	23%	71%	nd	0.30	34%	70%	nd	0.30	23%	70%	nd	0.30	24%	71%	nd	0.25	27%	70%	nd

Legend: *H.gam =Hanseniaspora gamundiae; H.vin= H.vineae; H.osm= H.osmophila; H.val= H.valbyensis; H.uva= H.uvarum; H.gui= H.guilliermondii; H.opu=Hopuntiae; L.lac =Kluyveromyces lactis; S.cer = Saccharomyces cerevisiae*

Kr, Alignment-free distance measure [53]

dDDH, Digital DNA:DNA hybridization [55]

ANI, Average Nucleotide Identity [54]

DDH, DNA-DNA homology values (data from Meyer et al. [53] and Cadez et al. [31])

Unrelated species

Closely related species

Conspecific strains

nd, not determined
