# Supplementary material for: Genomic content of a novel yeast species Hanseniaspora gamundiae sp. nov. from fungal stromata (Cyttaria) associated with a unique fermented beverage in Andean Patagonia, Argentina
Source: PLoS One. 2019 Jan 30;14(1):e0210792. doi: 10.1371/journal.pone.0210792 (PMC6353571; doi:10.1371/journal.pone.0210792)

S1 Fig. UPGMA dendrogram based on PCR fingerprints obtained with the microsatellite primers (GTG)5x and (ATG)5x on *Hanseniaspora gamundiae* sp. nov. and their closest relatives.

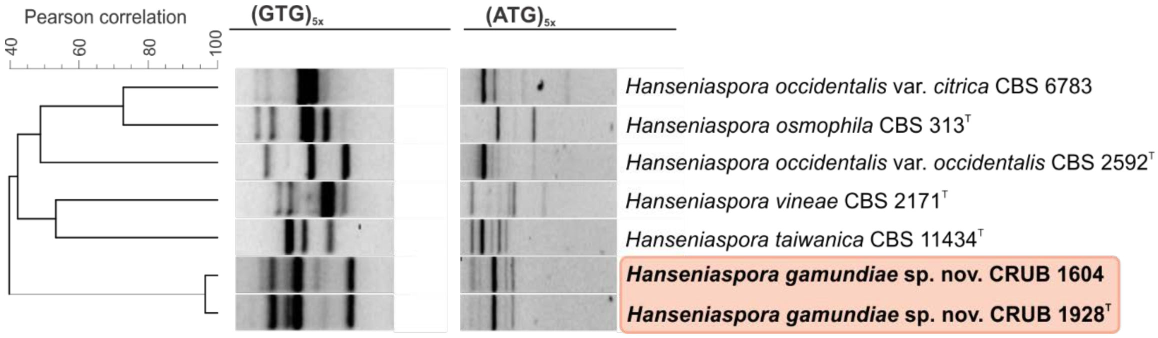

Supplement: S1 Fig — The distances between the strains were computed using Pearson's correlation coefficient. (PDF) [file pone.0210792.s003.pdf]
